# Broadband NIRS Cerebral Evaluation of the Hemodynamic and Oxidative State of Cytochrome-c-Oxidase Responses to +Gz Acceleration in Healthy Volunteers

**DOI:** 10.1007/978-3-030-34461-0_43

**Published:** 2020-01-01

**Authors:** F. Lange, G. Bale, P. Kaynezhad, R. D. Pollock, A. Stevenson, I. Tachtsidis

**Affiliations:** Department of Medical Physics and Biomedical Engineering, University College London, London, UK; The Human Performance Group, QinetiQ PLC, Farnborough, UK ; The Centre for Human and Applied Physiological Sciences, King’s College London, London, UK; The Human Performance Group, QinetiQ PLC, Farnborough, UK; Department of Medical Physics and Biomedical Engineering, University College London, London, UK

**Keywords:** NIRS, Broadband, Cytochrome-c-oxidase, Ischaemia, Human centrifuge

## Abstract

We used a miniature broadband NIRS system to monitor concentration changes in brain oxygenation (oxy- and deoxy- haemoglobin [HbO_2_], [HHb]) and oxidised cytochrome-c-oxidase ([oxCCO]) during a high +Gz acceleration, induced by a human centrifuge, on two healthy experienced volunteers (2 male, 34 and 37 years). We performed a sequence of several +Gz exposures that were terminated at the onset of visual symptoms (loss of peripheral vision). Systemic parameters were recorded (i.e. heart rate, blood pressure and arterial saturation), and brain tissue blood volume changes ([HbT] = [HbO_2_] + [HHb]) and oxygen delivery ([HbDiff] = [HbO_2_] - [HHb]) were calculated. Volunteer 1 demonstrated a decrease in [HbT] of −3.49 ± 0.02 μMol and [HbDiff] of −3.23 ± 0.44 μMol, and an increase of [oxCCO] of 0.42 ± 0.01μMol. Volunteer 2 demonstrated a decrease in [HbDiff] of −4.37 ± 0.23 μMol, and no significant change in [HbT] (0.53 ± 0.06 μMol) and [oxCCO] (0.09 ± 0.06 μMol). The variability of the brain metabolic response was related to the level of ischaemia, suggesting that suppression of metabolism was due to lack of glucose substrate delivery rather than oxygen availability.

## 1 Introduction

Near-infrared spectroscopy (NIRS) is now a common tool for monitoring non-invasively the tissue in-vivo changes in oxy- and deoxy haemoglobin ([HbO_2_] [HHb]) [1], and has been extensively used to monitor the function and physiology of the brain [2, 3].

Most NIRS systems use simple instrumentation and only measure the change in attenuation of 2 or 3 wavelengths, which is enough to calculate the changes in [HbO_2_] and [HHb]. Recent developments in NIRS instrumentation enables measurement of the change in attenuation of more than a hundred wavelengths [4, 5]. This technique is called broadband NIRS and can be used to retrieve information of a third chromophore, cytochrome-c-oxidase (CCO). CCO is the terminal electron acceptor of the electron transport chain in the mitochondria. Broadband NIRS monitors the changes in the redox state of CCO using the difference between the oxidised and reduced CCO spectra ([oxCCO]), for further information on this topic see the review by Bale et al. [6]. CCO provides information about metabolism, and in particular mitochondrial oxygenation. For example, it has been demonstrated that broadband NIRS has the potential to provide a real-time assessment of brain metabolism during neonatal hypoxic-ischaemic encephalopathy (HIE), and that oxCCO could be a clinically relevant metabolic marker [7].

In recent years, NIRS has also been used to monitor cerebral cortical oxygenation during +Gz exposure [8]. The goal of the present study is to assess the feasibility of monitoring haemodynamic and CCO responses during +Gz exposure using a broadband NIRS system. This possibility could improve the understanding of +Gz-induced loss of consciousness (G-LOC) and also provide a framework to investigate the mechanisms of cerebral hypoxic-ischaemia (HI) in healthy volunteers.

## 2 Methods

Two healthy non-pilot male volunteers, one aged 34 years (height 1.79 m and mass 75 kg) and one aged 37 years (height 1.82 m and mass 95 kg), participated in this preliminary study. The study was conducted under an ethical approved centrifuge procedure (Approval number TI1501625).

Acceleration profiles were generated using a 9.4-m-radius human centrifuge (QinetiQ, Farnborough, UK).

Volunteer 1 performed 2 centrifuge exposures consisting of a gradual increase (0.1G.s^−1^) in +Gz until the subject terminated the centrifuge run when peripheral light loss occurred. Each run was separated by 5 min of rest. Volunteer 2 performed 3 centrifuge exposures of these events.

Broadband NIRS measurements were made using an in-house developed miniature broadband system called miniCYRIL (CYtochrome Research Instrument and appLication) [9]. The system consists of a miniature Ocean Optics HL2000 white light source using a 20 W halogen-tungsten lamp and a customised Ocean Optics Ventana VIS-NIR miniature spectrometer. The miniCYRIL is a single channel system and the optodes were positioned over the left forehead with a source-detector separation of 3 cm, and secured with a headband and a custom 3D printed probe holder. It is worth mentioning that the probe holder was positioned with sufficient pressure to cause local blanching. Hence, we don’t expect any response from the skin vasculature [13]. Light intensity spectra from 600-1100 nm were collected at 1 Hz. The changes in concentration in [HbO_2_], [HHb] and [oxCCO] were calculated from the changes in attenuation from 780 to 900 nm using the UCLn algorithm [10]. Then, the change in concentration in total haemoglobin ([HbT]), which gives an indication of the blood volume changes, was calculated as [HbT] = [HbO_2_] + [HHb]. Finally, the change in the oxygen delivery ([HbDiff]) was calculated as [HbDiff] = [HbO_2_] - [HHb].

Systemic parameters were also recorded synchronously with the broadband NIRS data. Heart rate (HR) was recorded using a standard three-lead ECG from a proprietary system (locally manufactured) and beat-to-beat blood pressure (BP) from a Finapres device (Ohmeda). As we are interested in the brain’s mean BP (BMBP), we computed an estimation of the BMBP based on the measurement at the heart level mean BP (HLMBP) as expressed in reference [11] (i.e. BMBP = HLMBP – 19 mmHg*Gz). Arterial saturation (SaO_2_) was also recorded using a Masimo Rad-7 pulse oximeter with an ear probe (ear lobe). We observed a failure in the SaO_2_ measurement on volunteer 2, therefore this parameter was excluded from the analysis. Data are presented as mean and standard deviation (std).

## 3 Results

Figures 1 and 2 present the response of the NIRS and systemic data for each event for volunteers 1 and 2, respectively.

Figure 1 shows that volunteer 1 had an increased in HR of 45.6 ± 5.7 bpm and a drop in BMBP of −65.1 ± 8.5 mmHg. Regarding the NIRS measurement, volunteer 1 had an increase in [oxCCO] of 0.42 ± 0.01 μM, a decrease in [HbO_2_] of −3.36 ± 0.23 μM, [HbT] of −3.49 ± 0.02 μM and [HbDiff] of −3.23 ± 0.44 μM, and no meaningful change in [HHb] (−0.13 ± 0.21 μM). It is worth noting that volunteer 1 reported to be near G-LOC at the run termination time. Fig. 2 shows that volunteer 2 had an increased in HR of 40.7 ± 11.4 bpm and a drop in BMBP of −63.7 ± 6.4 mmHg. Regarding the NIRS measurement, volunteer 2 had no meaningful changes compared to volunteer 1 for [oxCCO] (0.09 ± 0.06 μM), an increase in [HHb] of 2.45 ± 0.14 μM, a decrease in [HbO_2_] of −1.92 ± 0.09 μM, and [HbDiff] of −4.37 ± 0.23 μM, and no significant change in [HbT] (0.53 ± 0.06 μM)

## 4 Discussion

A broadband NIRS system was used to evaluate the brain metabolic, oxygenation and haemodynamic responses during +Gz exposure. During these +Gz exposures, both volunteers experienced loss of vision (ischaemia of the retina), but volunteer 1 had a significant drop in [HbT] compared to volunteer 2. Also, both volunteers had similar reduction in brain oxygenation (drop in [HbDiff]) but volunteer 1 had a significant increase in [oxCCO] compared to volunteer 2.

Although some work investigating [oxCCO] in +Gz exposure was performed in the late 1980s [12–14], most NIRS studies focused only on the changes in brain oxygenation and haemodynamics to understand the mechanism of G-LOC. This is mainly due to the fact that the instrumentation required to be able to monitor this third chromophore is more complex and the interpretation of the [oxCCO] data can be difficult. However, the information provided from the current system can further elucidate the mechanism responsible for G-LOC. Thus, the development of a miniaturised system, such as used in the present study, provides new possibilities for the investigation of the interplay between brain oxygenation and metabolism in the high Gz environment.

The majority of the NIRS [oxCCO] contrast comes from the Copper A (CuA) centre in CCO. As CuA is a one-electron acceptor, changes in its redox state can be achieved by changing the rates of electron transfer to it or away from it. In HI, absence of oxygen prevents electron transfer from CuA, causing a reduction; additionally, a decrease of electrons to the chain will cause oxidation. Thus, changes in [oxCCO] reflect changes in (1) the supply of reducing equivalents to the respiratory chain; (2) the adenosine diphosphate (ADP) concentration, and (3) the cellular oxygen concentration [15].

It has been shown that HI produces a significant reduction in [oxCCO] in animal models [16, 17], due to oxygen depletion. However, it has also been shown in anaesthetised animals that only significant levels of hypoxia cause a reduction in oxCCO[18]. Drury et al. showed that in asphyxia in foetal sheep (umbilical cord occlusion), an increase in [oxCCO] was observed that was dependent on gestational age, with the more mature foetuses demonstrating a later reduction in [oxCCO] [19].

In the present study, the increase in [oxCCO] observed in volunteer 1 is thus likely to reflect reduction in (1) the supply of reducing equivalents to the respiratory chain; and (2) the ADP concentration. This suggests that G-LOC may, in part, be driven by a lack of sufficient delivery of glucose substrate to the mitochondrial respiratory chain, rather than an absence of oxygen. It is worth mentioning that this hypothesis holds for an ischemic non-hypoxic volunteer and that further studies will be needed to investigate the effect of hypoxic-ischemic events.

In conclusion, we have demonstrated the feasibility of using a miniaturised broadband NIRS system in a centrifuge, which unlocks new possibilities for studying G-LOC and the effects of HI in healthy non-anaesthetised volunteers. In our two case studies, we found differences in the metabolic response related to the level of brain tissue ischaemia, which suggests that the delivery of glucose substrate affects metabolism more than it does oxygen. These preliminary findings also suggest that a human centrifuge could be used as a model to study HI in healthy volunteers in order to better understand the underlying physiology of the condition.

## Figures and Tables

**Figure 1:**
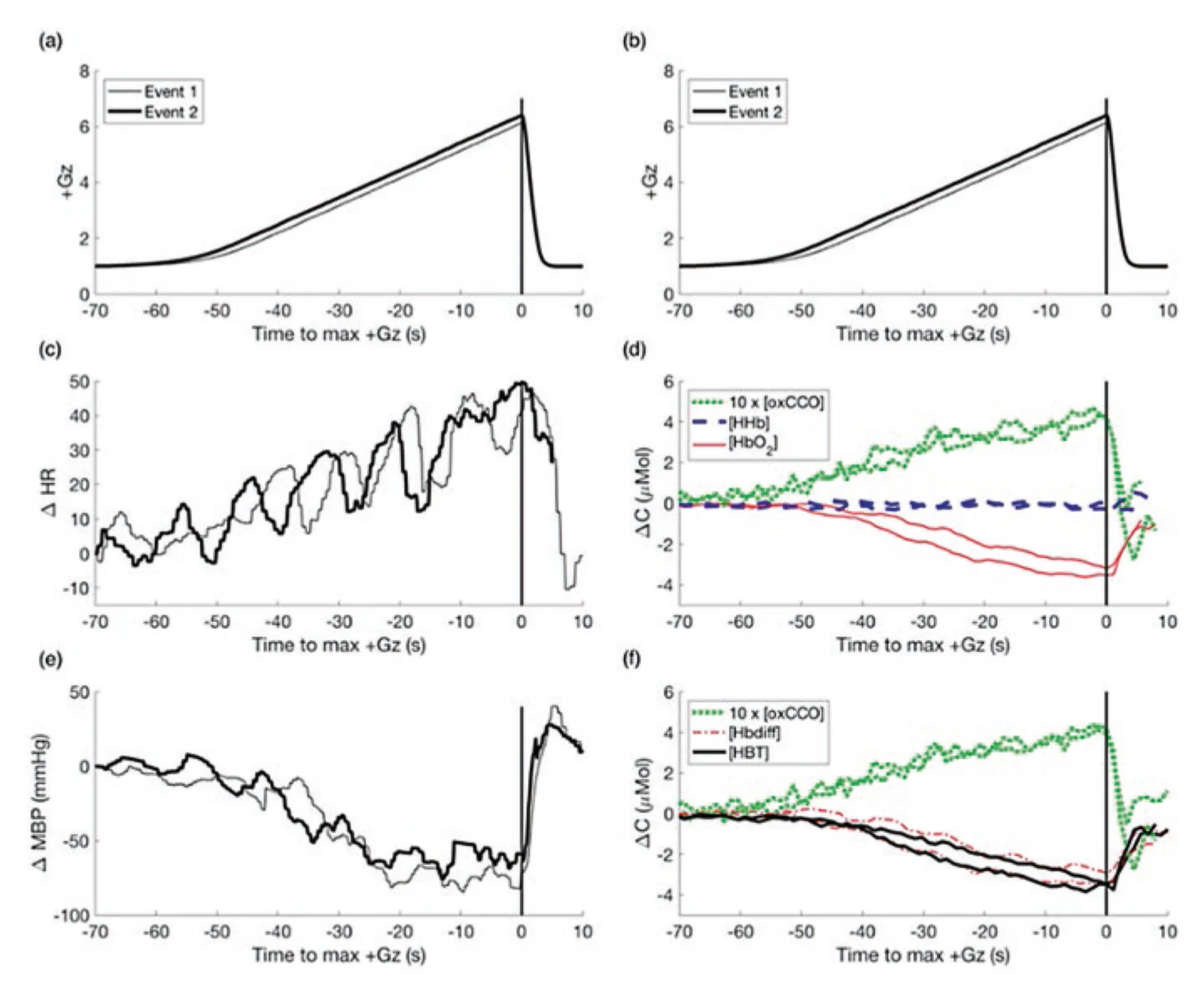
NIRS and systemic responses to the +Gz events for volunteer 1. (**a**) and (**b**) present the +Gz events dynamic events and are repeated to help the visualisation of the systemic data (left panel) and NIRS data (right panel). (**c**) presents the changes in heart rate (HR). (**d**) presents the changes in [HbO_2_] (solid red line) [HHb] (dashed blue lines), and [oxCCO] (scaled by a factor 10) (dotted green lines). (**e**) presents the changes of mean blood pressure (MBP) at head level. (**f**) presents the changes in [HbT] (solid black lines) and [HbDiff] together with the changes in [oxCCO] (scaled by a factor of 10) (dotted green lines). On all panels, the vertical black line presents the time of maximum +Gz

**Figure 2:**
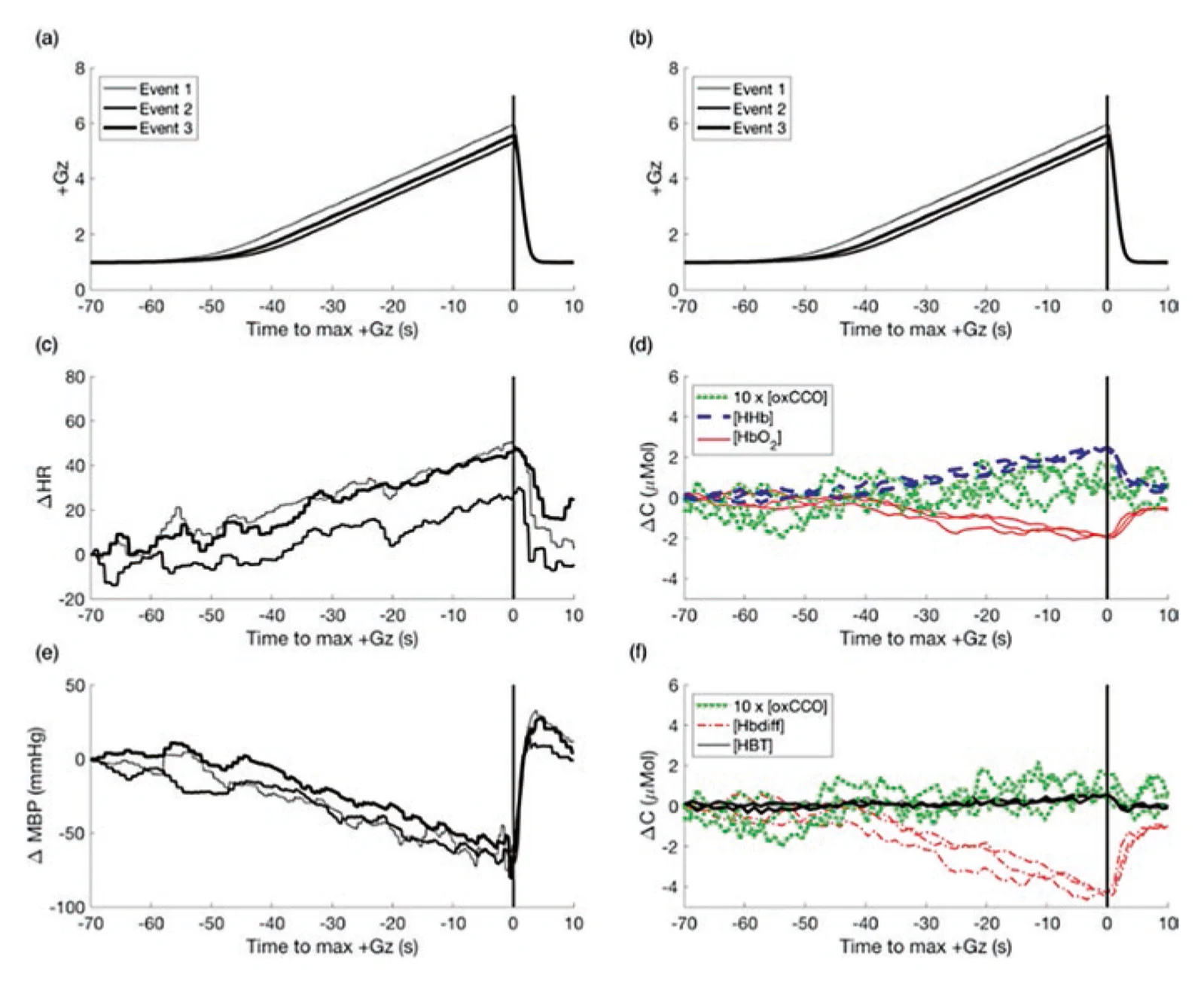
NIRS and systemic responses to the +Gz events for volunteer 2. (**a**) and (**b**) present the +Gz events dynamic events and are repeated to help the visualisation of the systemic data (left panel) and NIRS data (right panel). (**c**) presents the changes in heart rate (HR). (**d**) presents the changes in [HbO_2_] (solid red line) [HHb] (dashed blue lines), and [oxCCO] (scaled by a factor 10) (dotted green lines). (**e**) presents the changes of mean blood pressure (MBP) at head level. (**f**) presents the changes in [HbT] (solid black lines) and [HbDiff] together with the changes in [oxCCO] (scaled by a factor of 10) (dotted green lines). On all panels, the vertical black line presents the time of maximum +Gz
